# CPU-GPU hybrid accelerating the Zuker algorithm for RNA secondary structure prediction applications

**DOI:** 10.1186/1471-2164-13-S1-S14

**Published:** 2012-01-17

**Authors:** Guoqing Lei, Yong Dou, Wen Wan, Fei Xia, Rongchun Li, Meng Ma, Dan Zou

**Affiliations:** 1 National Laboratory for Parallel & Distributed Processing, Department of Computer Science, National University of Defense Technology, Changsha, 410073, China

## Abstract

**Background:**

Prediction of ribonucleic acid (RNA) secondary structure remains one of the most important research areas in bioinformatics. The Zuker algorithm is one of the most popular methods of free energy minimization for RNA secondary structure prediction. Thus far, few studies have been reported on the acceleration of the Zuker algorithm on general-purpose processors or on extra accelerators such as Field Programmable Gate-Array (FPGA) and Graphics Processing Units (GPU). To the best of our knowledge, no implementation combines both CPU and extra accelerators, such as GPUs, to accelerate the Zuker algorithm applications.

**Results:**

In this paper, a CPU-GPU hybrid computing system that accelerates Zuker algorithm applications for RNA secondary structure prediction is proposed. The computing tasks are allocated between CPU and GPU for parallel cooperate execution. Performance differences between the CPU and the GPU in the task-allocation scheme are considered to obtain workload balance. To improve the hybrid system performance, the Zuker algorithm is optimally implemented with special methods for CPU and GPU architecture.

**Conclusions:**

Speedup of 15.93× over optimized multi-core SIMD CPU implementation and performance advantage of 16% over optimized GPU implementation are shown in the experimental results. More than 14% of the sequences are executed on CPU in the hybrid system. The system combining CPU and GPU to accelerate the Zuker algorithm is proven to be promising and can be applied to other bioinformatics applications.

## Introduction

RNA is an important molecule in biological systems. The function of RNA can be derived generally from its secondary structure. Recently, computational and mathematical methods such as thermodynamic energy minimization, homologous comparative sequences, and stochastic context-free grammar methods have been widely used to predict the RNA secondary structure. Among these, the Zuker algorithm is the most popularly and widely used free energy minimization algorithm for RNA secondary structure prediction [1]. The Zuker algorithm was first presented in 1981 by M. Zuker. Its time complexity is O(*n*^4^) [2], and its spatial complexity is O(*n*^2^), where N is the length of the sequence. The optimized algorithm [3] reduces the time complexity to O(*n*^3^) by limiting the length of interior loop size. This algorithm is commonly used in many popular secondary structure prediction software, such as Mfold and ViennaPackage. The Zuker algorithm is able to predict short sequences because accuracy is decreased rapidly as the sequence grows longer [1]. However, the exponential increment of RNA sequences makes conventional computers incapable of meeting the demand of multiple-sequence processes. For instance, a single-core Intel Xeon E5620 processor consumes 18 ms to predict the secondary structure of a single RNA sequence with length 120, and needs over 370s for 20000 RNA sequences of the same length. Execution time increases rapidly as sequence number grows.

Recently, heterogeneous computing systems have been broadly used in high-performance computing. Three of the first five Top 500 supercomputers are built based on CPU-GPU heterogeneous architecture [4]. Specifically, Tian-1A, the first supercomputer in 2010's Top 500, is the first to exploit the heterogeneous system. The computing method on the CPU-GPU heterogeneous system has been proven effective in HPC, and its performance has grown rapidly with improvements of CPU and GPU technology.

The availability of multiple cores on a modern processor chip makes the CPU a more powerful computing platform for general applications. Parallel programming methods on multi-core CPUs, such as POSIX Thread (pThread) library [5], OpenMP library [6], and Intel Threading Building Blocks [7], effectively simplify concurrent software development on these platforms.

To date, GPUs have emerged as favorable and popular accelerator devices that keep up with the increasing demands of the gaming industry. GPUs have developed into more general, highly parallel, multiple-core processing architecture to keep up with the demands of the computer games, which increase faster than processor clock speeds. A number of libraries have been developed to allow users to write non-graphic applications computed on GPUs, which is known as General Purpose computation on GPUs (GPGPU). The development of GPGPU libraries, such as the NVidia CUDA [8], BrookGPU [9], the ATI Stream SDK [10], Sh [11], and OpenCL [12] have made GPGPU applications increasingly easy to develop. With the rapidly improving computational capability of CPU and GPU, exploring the performance potentials of both systems has become a problem. In the current heterogeneous system, CPU plays as program controller while GPU performs most of the computing task. The performance of CPU has not yet been fully explored. In the present study, a CPU-GPU hybrid system that efficiently explores both CPU and GPU computing potentials is presented. A part of the computing tasks of the GPU is allocated for the CPU, reducing the performance loss from waiting for results from the former. Speedup factor of 6.75× over optimized multi-core SIMD CPU implementation and performance improvement factor of 16% over optimized GPU implementation are shown in the experimental results. In addition, more than 14% of sequences are computed on CPU in the hybrid system.

## Related works

Accelerating or parallelizing the Zuker algorithm for RNA secondary structure prediction on modern computing platforms is not new. Tan et al. [13] reported 8 speedup on a cluster with 16 Opteron processors that for cluster parallel computers. On shared memory parallel computers, Tan et al. [14] reported 19× speedup on a 32-processor system, DAWNING 4000. Mathuriya et al. [15] present their parallel implementation of GTfold on a 32-core IBM P5-570 server and 19.8 speedup is achieved. On multi-core processor, Wu et al. [16] parallelized the RNAfold program on quad-core Intel Q6600 processor to obtain 3.88 speedup. Another solution to accelerate the Zuker algorithm is to use accelerators. Based on the FPGA platform, Dou et al. [17] and Jacob et al. [18] presented fine-grain parallel implementation and one to two orders of magnitude speedup over the general-purpose processor. Based on the GPU platform, G. Rizk et al. [19] accelerated the Unafold application to attain 33.1 speedup over a single-core Xeon GPU. The problem of accelerating the Zuker algorithm or other applications on large-scale parallel computers is that use, maintenance, and management costs are very high. High-performance parallel computers are too expensive for use by many research institutes. Although specialized coprocessing accelerators are able to achieve high performance, the computing capability of CPUs in systems where the CPU is the program controller has not been explored. A heterogeneous hybrid CPU-GPU computing scheme would be capable of not only exploiting the power of accelerating device, but also of exploring the CPU computing potential. To our best knowledge, no parallel implementation for accelerating Zuker algorithm on CPU-GPU hybrid system has thus far been realized.

## Background

### Overview of the Zuker algorithm

The Zuker algorithm predicts the most stable secondary structure for a single RNA sequence by computing its minimal free energy (MFE). It uses a "nearest neighbor" model and empirical estimates of thermodynamic parameters for neighboring interactions and loop entropies to score all possible structures [20]. The main idea is that the secondary structure of an RNA sequence consists of four fundamental substructures: stack, hairpin, internal loop, and multi-branched loop. These fundamental substructures are independent of one another, and the energy of a secondary structure is assumed to be the sum of the substructure energies. With a single RNA sequence as input, the algorithm is executed in two steps. First, it calculates the minimal free energy of the input RNA sequence on a group of recurrence relations, as shown in Formula (1) to (5). Second, it performs a trace-back to recover the secondary structure with the base pairs. Experiments show that the first step consumes more than 99% of the total execution time. Thus, computing energy matrices as quickly as possible is critical to improve the performance.

(1) W(j)= min{W(j-1),min[V(i,j)+W(i-1)]}

(2) $$V\left(i,j\right)=\left\{\begin{array}{cc} \min\left\{\begin{array}{c} eH\left(i,j\right)\\ eS\left(i,j\right)+V\left(i+1,j-1\right)\\ VBI\left(i,j\right)\\ VM\left(i,j\right) \end{array}\right) & pair\;\left(i,j\right)\;is\;allowed\\ \infty & pair\;\left(i,j\right)\;is\;not\;allowed \end{array}\right)$$

(3) $$VBI\left(i,j\right)=\underset{i<k<l<j}{\min}\left\{eL\left(i,j,k,l\right)+V\left(k,l\right)\right\}$$

(4) $$VM\left(i,j\right)=\underset{i<k<j}{\min}\left\{WM\left(i,k\right)+WM\left(k+1,j\right)\right\}$$

(5) WM(i,j)= min{VM(i,j),min[WM(i+1,j),WM(i,j-1)],V(i,j)}

Suppose *r*_1_*r*_2_...*r*_*i*_...*r*_*j*_...*r*_*n *_represents an RNA sequence where i and j are the location of the nucleotides in the sequence, and n is the sequence length. Formula (1) to (5) describe the method for computing free energy. Here, W(j) is the energy of an optimal structure for the subsequence *r*_1_*r*_2 _...... *r*_*j*_; V(i, j) is the energy of the optimal structure of the subsequence *r*_*i*_*r*_*i*+1_...*r*_*j*_; VBI(i, j) is the energy of the subsequence *r*_*i *_through *r*_*j*_, where *r*_*i*_*r*_*j *_closes a bulge or an internal loop; VM(i, j) is the energy of the subsequence *r*_*i *_through *r*_*j*_, where *r*_*i*_*r*_*j *_closes a multi-branched loop; and eS(i, j), eH(i, j), and eL(i, j, k, l) are free energy functions used to compute the energy of stacked pair, hairpin loop, and internal loop respectively. Given any subsequence *r*_*i*_...*r*_*j*_, the Zuker algorithm calculates free energies of the four possible substructures if pair (i,j) is allowed. The results correspond to the four items in Formula (2): eH(i, j), *eS*(*i,j*) + *V*(*i *+ 1, *j *- 1), VBI(i, j), and VM(i, j). The Zuker algorithm then selects the minimum value V(i,j) among the four results. The subsequence grows from *r*_1_, *r*_1_*r*_2_, ..., *r*_1_*r*_2_....*r*_*j*-1 _to *r*_1_*r*_2_...*r*_*i*_...*r*_*j*_...*r*_*n*_. The lowest conformational free energy is stored in vector W. The corresponding energy of *r*_1 _is stored in W(1), and *r*_1_*r*_2 _is stored in W(2), and so on for longer fragments, such as W(j-1) for *r*_1_*r*_2_*r*_3 _...... *r*_*j*-1_. Once the longest fragment (i.e., the complete sequence) is considered, the lowest conformational free energy of whole RNA sequence is calculated, and the energy of the most energetically stable structure is contained in W(n). The corresponding secondary structure is then obtained by a trace-back procedure from the vector W, and matrices V and WM.

### Overview of CPU architecture

**Figure 1:**
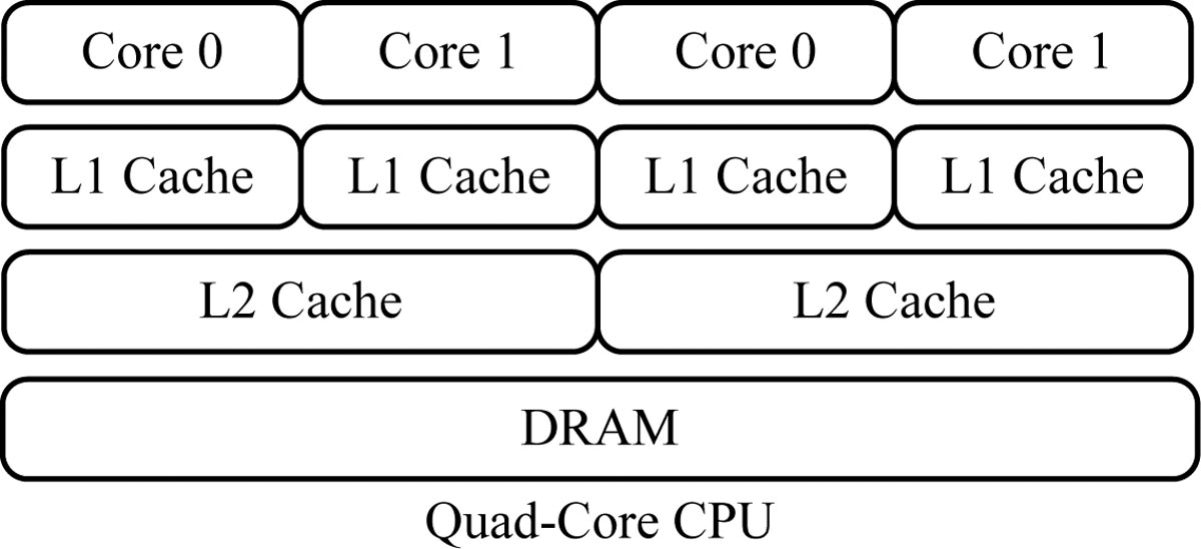
**Quad-core CPU architecture.** This figure describes the architecture of quad-core CPU. Each core owns L1 cache, and two cores shared L2 caches.

In recent years, the number of processing cores available on a modern processor chip has increased steadily. Quad-core CPUs are now the norm, and more core systems have become economically available. Figure 1 shows a typical quad-core CPU architecture. Each core hosts one thread at a time, with a set of registers containing thread state and a large functional unit devoted to computation and management.

Multi-core CPUs make rethinking the development of application software necessary. Application programmers should explicitly use concurrency to approximate the peak performance of modern processors. To utilize all available processing power of these processors, computationally intensive tasks should be split up into subtasks for execution on different cores. A number of different approaches are available for parallel programming on multi-core CPUs, ranging from low-level multi-tasking or multi-threading such as POSIX Thread (pThread) library [5], over high-level libraries, such as Intel Threading Building Blocks [7], which provide certain abstractions and features attempting to simplify concurrent software development, to programming languages or language extensions developed specifically for concurrency, such as OpenMP [6]. Apart from multi-threading parallelism on the multi-core platform, data parallelism can be explored by SIMD vector processing instructions. For example, Intel has an SIMD instruction set called streaming SIMD extensions (SSE) [21]. SSE contains 70 new instructions and 8 new 128-bit registers. SSE2 adds new arithmetic types, including maximum and minimum operations. Each 128-bit register can be partitioned to perform four 32-bit integers, or single-precision floating points, or eight 16-bit short integers, or sixteen 8-bit bytes operations in parallel.

### Overview of GPU architecture

**Figure 2:**
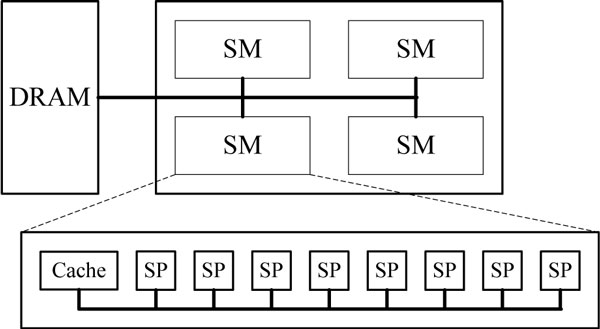
**Nvidia GPU architecture.** This figure describes the architecture of Nvidia GPU. It is composed of four streaming multi-processors, each containing eight scalar processors. This architecture can be easily extended to contain more multi-processors.

Figure 2 depicts the GPU architecture from Nvidia. The GPU contains a scalable array of multi-threaded processing units known as streaming multi-processors (SMs). Each SM contains eight scalar processor (SP) cores that execute actual instructions. Each SM performs computation independently; however, SP cores within the single multi-processor execute instructions synchronously. This paradigm, called "single instruction, multiple threads" (SIMT) [8], is the basic computing scheme of GPU. Threads are grouped into blocks, and multiple blocks may run in a grid of blocks. Such structured sets of threads may be launched on a kernel of code for data processing in the device memory. Threads of the same block share data on-chip memory, and coordinate through synchronization points. CUDA is the most used NVIDIA parallel programming model and software environment for running applications on GPUs. CUDA abstracts the architecture to parallel programmers via simple extensions to C programming language. In CUDA, threads in one block are created, managed, scheduled, and executed in a unit called Warp using a combination of 32 threads with consecutive thread ID. Parallel performance is improved when all threads in the same Warp follow the same execution path.

A hierarchy of GPU memory architecture is available for programmers to utilize. The fastest memories are the shared memory and registers with severely limited sizes. Registers are allocated by a compiler, whereas shared memory is allocated by a programmer. The constant, texture, and global memory are all located on the off-chip DRAM. The texture and constant memory are read-only and are cached. The global memory is the slowest memory, and its access may take hundreds of clock cycles.

Most research in GPU programming involves finding the optimal way to solve a problem on data-parallel architecture while best using optimizations specific to GPU architectures. Of the many GPGPU APIs available [8-11], the Nvidia CUDA stands out as the most developed and advanced. GPGPU API only operates on Nvidia GPUs. Our development of GPGPU applications uses CUDA API limited to Nvidia graphics cards.

## CPU-GPU hybrid computing system

### System architecture

**Figure 3:**
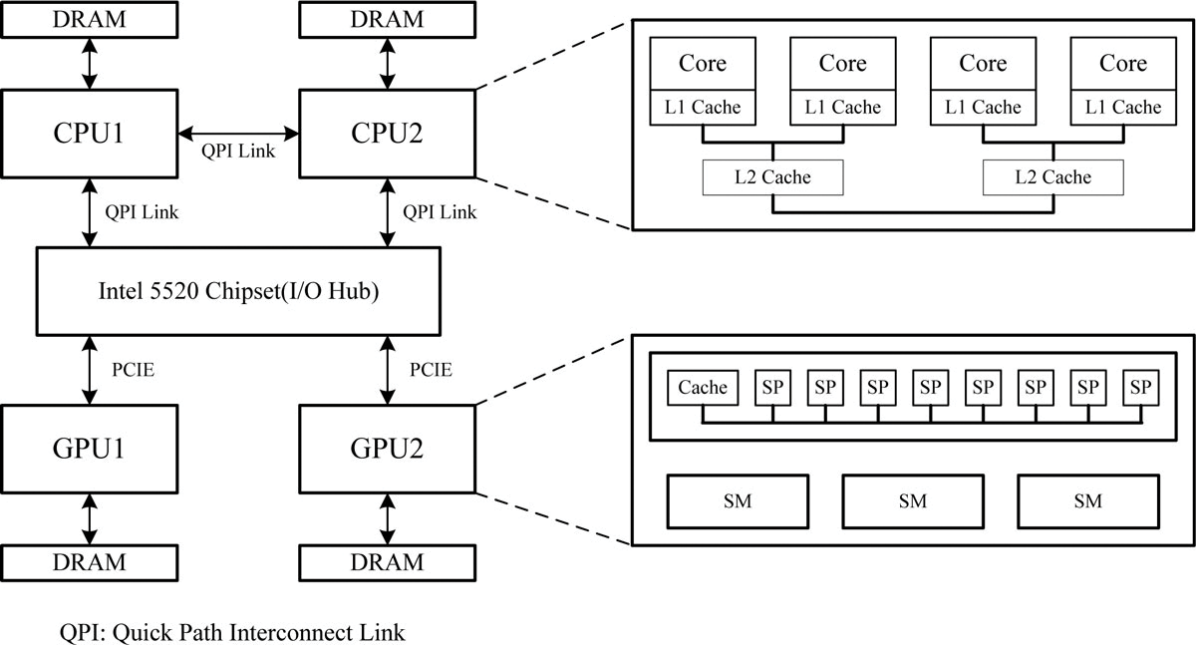
**CPU-GPU hybrid accelerating system architecture.** This figure describes the architecture of our CPU-GPU hybrid accelerating system with two CPUs and GPUs. Both CPUs and GPUs have their own storage. As the GPU performs computation, the CPU simultaneously performs computing tasks, instead of waiting for energy results from the GPU.

A CPU-GPU hybrid Zuker accelerating system is generally composed of several CPUs and GPUs. Figure 3 depicts the hybrid system architecture with two CPUs and two GPUs. The CPUs and GPUs communicate via I/O Hub Chipset, and are connected to I/O Hub by a Quick Path Interconnect Link (QPI) and PCIE. Both CPUs and GPUs have their own storage. Each CPU has four cores. Each core owns an L1 cache. Two cores share one L2 cache. The GPU has several SMs each with several SPs. The CPU is responsible for program control, including initiating the Zuker application, allocating tasks between CPU and GPU, initiating the GPU computation, reading the result from the GPU, and backtracking of energy matrices. The GPU is responsible for filling energy matrices for multiple RNA sequences. As the GPU performs computation, the CPU simultaneously performs computing tasks, instead of waiting for energy results from the GPU.

The hybrid accelerating system predicts secondary structures of all RNA sequences input. Firstly, the CPU reads all RNA sequences, and performs some preprocess operations such as memory allocation for energy matrices. After the task allocation, CPUs and GPUs fill energy matrices in parallel. The CPU receives results from the GPU device memory after energy filling is through. Finally, the CPU executes backtrack operations, and displays all the RNA secondary structure information to the user.

### Task-allocation and execution scheme

The proposed hybrid accelerating system fully exploits the performance potentials of both CPU and GPU to obtain higher system performance for the same computing task. The key factor in the hybrid accelerating system is the task allocation between the CPU and the GPU. Given several RNA sequences numbered 1,2,...,N, the task allocation obtains a boundary sequence number B. The sequences with number below B are to be computed on the CPU. The rest of sequences are computed on the GPU. To achieve load balancing in the task allocation, the processing capability of the CPU and the GPU for RNA sequence with different length is estimated beforehand.

**Figure 4:**
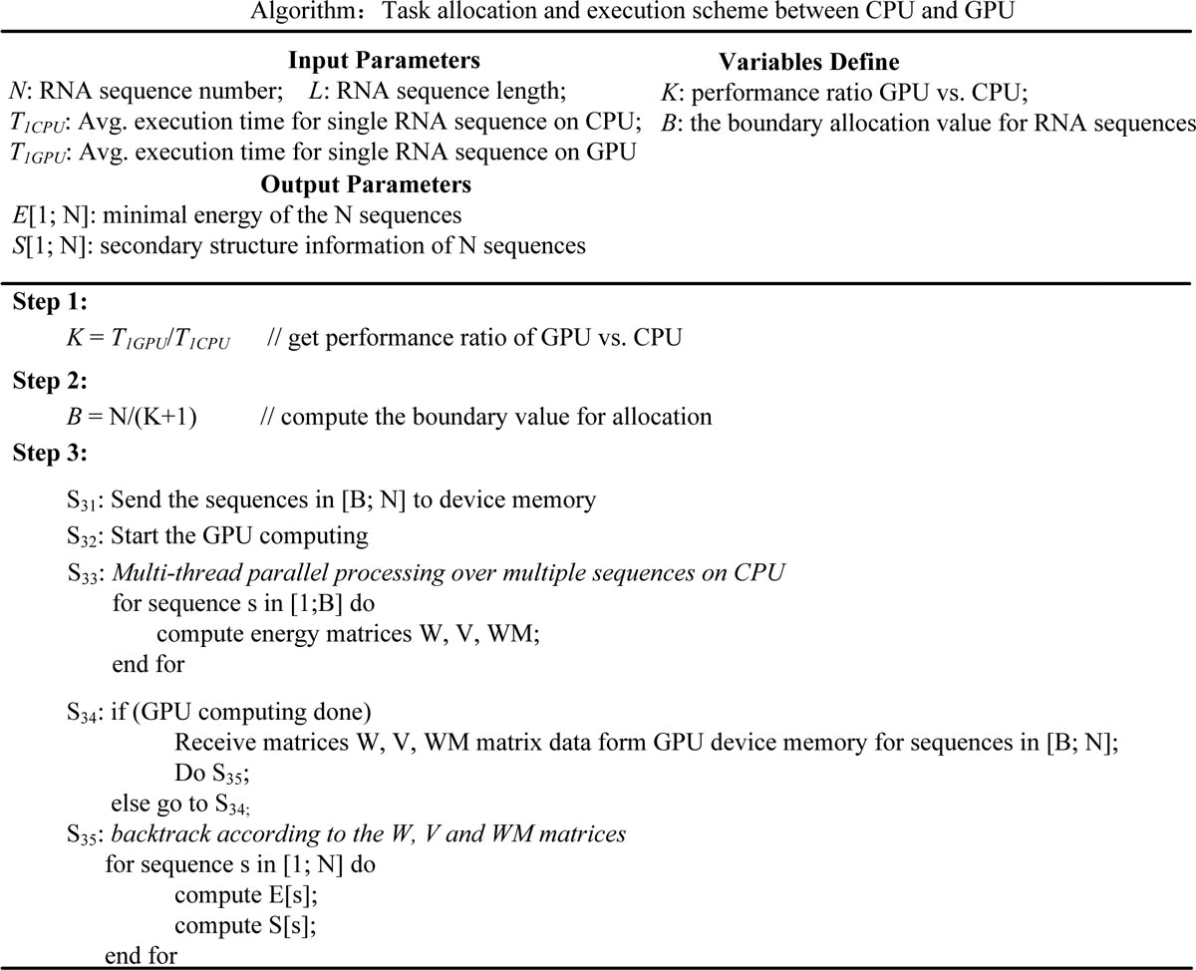
**Task-allocation and execution scheme between CPU and GPU.** This figure describes task allocation and execution scheme of our CPU-GPU hybrid accelerating system.

Figure 4 demonstrates the task-allocation and execution algorithm. The input of the algorithm is N sequences of length L, the estimated average execution time for a single sequence on CPU and GPU. The output is minimal energy and secondary structure information of N sequences. The task allocation algorithm comprises three steps. In Step 1, GPU vs. CPU speedup based on the average execution time is calculated. The boundary allocation value B is then computed in Step 2. Finally, in Step 3, the entire hybrid computing scheme is demonstrated. In this step, the CPU begins the computing as soon as the sequences in [B: N] are sent to the GPU device. After the GPU is initiated, the sequences in [1: B] are processed by multiple threads parallel on CPU. Each thread is responsible for calculation of the energy matrices of the sequence scheduled by the Intel compiler for OpenMP library. After the CPU processing, data of the energy matrices on the GPU are retrieved after GPU processing is completed. Finally, the CPU executes backtrack operations by reading all energy matrices of sequences [1: N], and produces minimal energy and structure information output for each sequence.

Calculation of the boundary sequence number B is demonstrated below. The average execution time of a single sequence on CPU and GPU are first assumed as *T*_1*CPU *_and *T*_1*GPU*_, respectively. The entire energy filling time will be minimal when the CPU and GPU executions are overlapped nearly completely, satisfying equation *T*_1*CPU *_· *B *= *T*_1*GPU *_· (*N *- *B*). The boundary value B then can be computed by equation $B=\frac{1}{K+1}\cdot N$, where K denotes the speedup of GPU vs. CPU and equals $\frac{T_{1CPU}}{T_{1GPU}}$. The expression $\frac{1}{K+1}$ is called the allocation ratio.

## Performance tuning schemes

### Multi-core CPU implementation

Performance tuning on multi-core CPU mainly consists of compiler optimization, SSE, and multi-thread processing. First, compiler optimization is used by setting the -O2 option to improve the performance of original software. SSE instruction is then utilized to accelerate computation of the VM matrix. The element VM(i,j) depends on the row WM(i,*) and the column WM(*,j). Computation on VM(i,j) is divided into two steps. In the first step, elements on the row WM(i,*) are added to corresponding elements on the column WM(*,j). The minimal value of the sums is then chosen to be VM(i,j). Using SSE instructions, the four elements from row i and column j of matrix WM are read and stored in a 128-bit register. Two 128-bit registers are added, such that four simultaneous additions are performed to compute VM(i,j). The third approach for boosting performance of the CPU is by OpenMP libraries that explore parallel processing of multiple sequences. Energy matrices computations of different sequences are mapped onto different threads for processing by an Intel compiler for OpenMP libraries. Each thread is responsible for the matrices computations of a single sequence allocated onto it. Threads are working independently from each other to access their own memory spaces, achieving high coarse-grain parallel performance.

### GPU implementation

**Figure 5:**
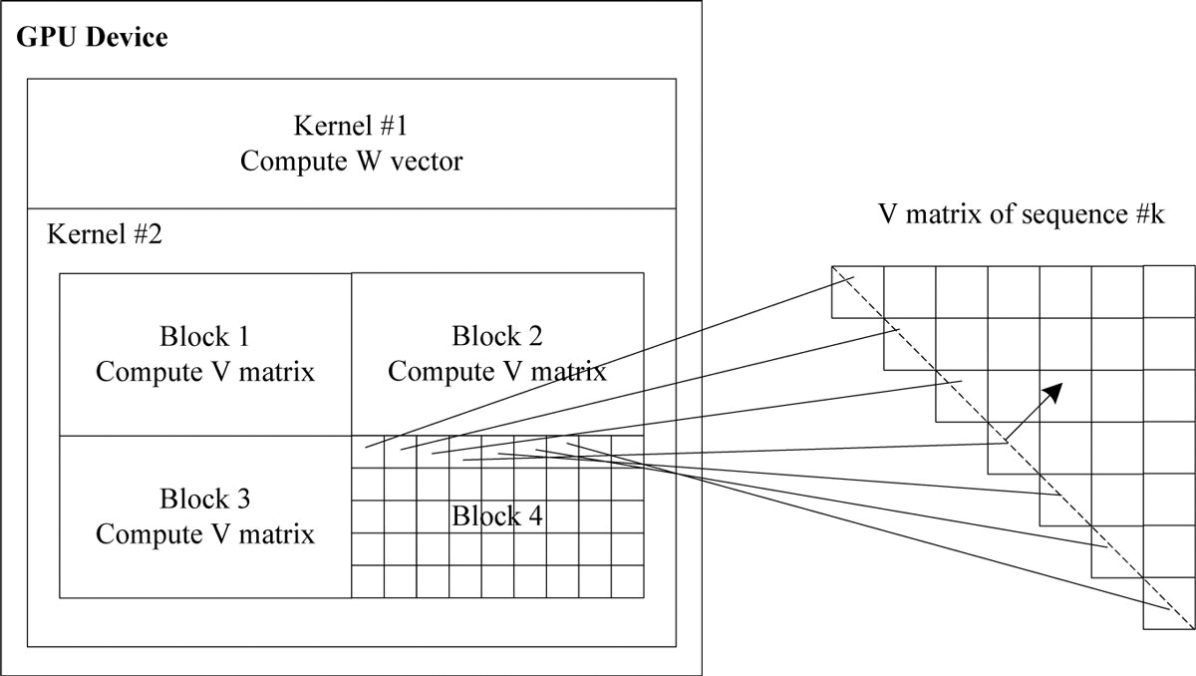
**Optimization scheme of hierarchy parallelism on GPU.** This figure describes the hierarchy parallelism optimization scheme on GPU. The hierarchy parallelism is composed of kernel-level, sequence-level and element-level respectively.

Several parallel schemes are used to improve the Zuker algorithm performance on GPU. Basically, hierarchy parallelism is adopted. The first layer utilizes kernel-level parallelism for execution of multiple concurrent kernels. In Figure 5, the GPU device consists of two kernels, ♯1 and ♯2, executing concurrently for W vector and V matrix computations. The second layer employs sequence-level parallelism, where sequences are processed by different blocks in the same kernel. One block can hold multiple sequences when the length of the sequence is sufficiently short. In Figure 5, the ♯2 kernel contains several blocks, with each block responsible for V matrix computation of one or more sequences. The last layer uses parallel execution of diagonal elements in a single sequence. In Figure 5, elements in the diagonal of V matrix are mapped onto different threads in a block for parallel execution.

Next, memory optimizations are adopted to fully use limited device memory. Data type transformation, immediate assignment, and redundant data cutting schemes are used to reduce parameters storage requirement in the device memory. To improve the memory access performance, column data of the matrix are stored in a consecutive memory address array. Sequence data and energy matrices are stored in shared memory and global memory respectively. A data reuse scheme is implemented by storing partly energy matrices in shared memory. Mediating data in the computation is partly stored in shared memory and registers to improve memory access and memory bandwidth.

Computation of the energy elements in the diagonal is mapped to the threads in a warp to explore fine-grain parallelism. Element computation in the V matrix is divided in two situations based on whether or not the *i*th and the *j*th nucleotide become a pair. Hence, execution path of the threads in the same warp may differ. In the proposed hybrid system, the matrix elements with the same execution path are mapped to the threads in a warp with consecutive thread ID to effectively improve the parallel efficiency. 

**Figure 6:**
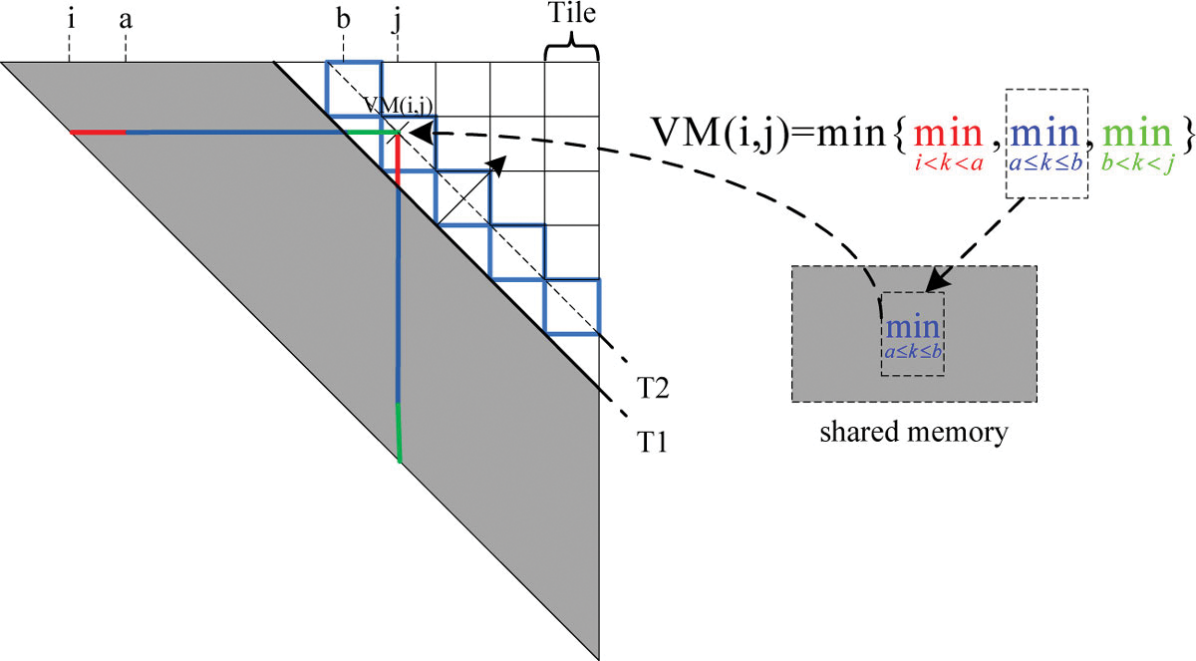
**Tiling scheme for VM matrix computations.** This figure describes the tiling scheme for VM matrix computations on GPU. All blue portions of VM elements in the tile blocks (border with blue color) are calculated ahead of time.

Finally, tiling method is used to accelerate computation of the VM matrix of each sequence. Figure 6 illustrates the computation of the VM matrix tiled into small blocks. The VM matrix elements in the diagonal are parallel computed in different threads. The computing direction is moving from the principal diagonal to the top-right. The shadow area represents the already known VM and WM matrix elements (shown in the same matrix). The VM(i,j) in a tile block, which depends on the elements in row WM(i,*) and column WM(*,j), is divided into three parts with different colors. From the already known WM matrix data (blue in the shadowed area), the blue portion of the VM(i,j) computation is calculated ahead of time and stored into the shared memory for fast access. All blue portions of VM elements in the tile blocks (border with blue color) are calculated ahead of time. When the computing diagonal moves from T1 to T2, the pre-calculated blue portion results are read directly from the shared memory to accelerate the computation of the elements in the diagonal of T2.

## Experiments and results

### Experimental environment

In general, the hybrid accelerating system is composed of multiple CPUs and GPUs. For ease, the proposed prototype system for performance evaluation only consists of a host PC and a GPU card. The host in the testbed is equipped with an Intel Xeon E5620 Quad 2.4 GHz CPU, 24 GB memory, and ASUS Z8PE-D12 Motherboard (Intel 5520 chipset running Windows 7 with Visual Studio 2010 development environment). Geforce GTX580 with CUDA toolkit 4.0 is utilized as the GPU experimental platform. The Zuker algorithm program RNAfold is derived from the software package ViennaRNA-1.8.4 [22].

### CPU performance

Four groups (A, B, C, D) of randomly generated RNA sequences are chosen. Each group consists of 1024 sequences of same length. The average execution time per sequence is measured by calculating energy matrices of all sequences in each group when performance-tuning method is adopted gradually from O2 compiler optimization, SSE, to multi-thread processing. The results are shown in Table 1.

**Table 1:** Average execution time (ms) per sequence, and speedup (Sp) for four groups of sequences when different performance tuning method is adopted gradually on CPU

Opt. methods	A (L = 68)		B (L = 120)		C (L = 154)		D (L = 221)	
	
	Time	Sp.	Time	Sp.	Time	Sp.	Time	Sp.
None	7.449	1	35.282	1	66.270	1	155.954	1
O2	3.930	1.90	18.631	1.89	34.795	1.90	82.266	1.90
SSE2	4.052	1.84	19.164	1.84	34.901	1.90	80.177	1.95
Multi-thread	1.081	6.89	4.737	7.45	8.424	7.87	19.059	8.18

An average 1.90× speedup over the naive implementation on single Xeon E5620 core can be achieved in the O2 optimization. The SSE2 method is awkward for the first three groups of sequences, the control penalty induced is over the original VM decomposition operations for short sequence processes. After the multi-thread processing, the highest performance has been obtained for each group.

### GPU performance

Table 2 shows the average execution time per RNA sequence for different groups when different performance tuning methods are adopted gradually on GPU. Sequences in the groups A, B, C, D are randomly generated with 1200 sequences of similar length. Hierarchy parallelism execution on GPU is chosen as the baseline. For each group, gradual adoption of the different optimization methods significantly improves the performance.

**Table 2:** Average execution time (ms) per sequence and speedup (Sp) for four sequence groups when different performance tuning methods are adopted gradually on GPU

Opt. methods	A (L = 68)		B (L = 120)		C (L = 154)		D (L = 221)	
	
	Time	Sp.	Time	Sp.	Time	Sp.	Time	Sp.
Hierarchy parallelism	0.452	1	2.402	1	3.530	1	20.067	1
Memory optimization	0.199	2.27	0.855	2.81	1.561	2.26	8.876	2.26
Thread scheduling and tiling	0.068	6.65	0.293	8.20	0.754	4.68	3.270	6.14

After thread scheduling and tiling, the optimal average execution time for each sequence of the corresponding group is available. The average execution time for the sequence with length 120 on a single GTX 280 card is inferred as 0.473 ms [19]; however, it is 0.293 ms on the proposed method. Thus, a 1.61× speedup can be achieved over single GTX 280 card implementation.

### Hybrid system performance

Four groups of randomly generated RNA sequences are tested on our hybrid system. Each group contains 20000 sequences of similar length. Sequence lengths in the four groups are 68, 120, 154, and 221 respectively. The average estimated execution time for single sequence of each group is demonstrated in Tables 1 and 2. For each group, GPU vs. CPU speedup is calculated from the average execution time on CPU and GPU. The allocation ratio is further calculated by GPU vs. CPU speedup. The results are shown in Table 3.

**Table 3:** Experimental results of the estimated task allocation ratio for different sequence groups

	A (L = 68)	B (L = 120)	C (L = 154)	D (L = 221)
Avg. CPU exe. time	1.081	4.737	8.424	19.059
Avg. GPU exe. time	0.068	0.293	0.754	3.270
Speedup GPU vs. CPU	15.90	16.17	11.17	5.83
Estimated allocation ratio	5.92%	5.83%	8.22%	14.64%

#### Allocation scheme evaluation

**Figure 7:**
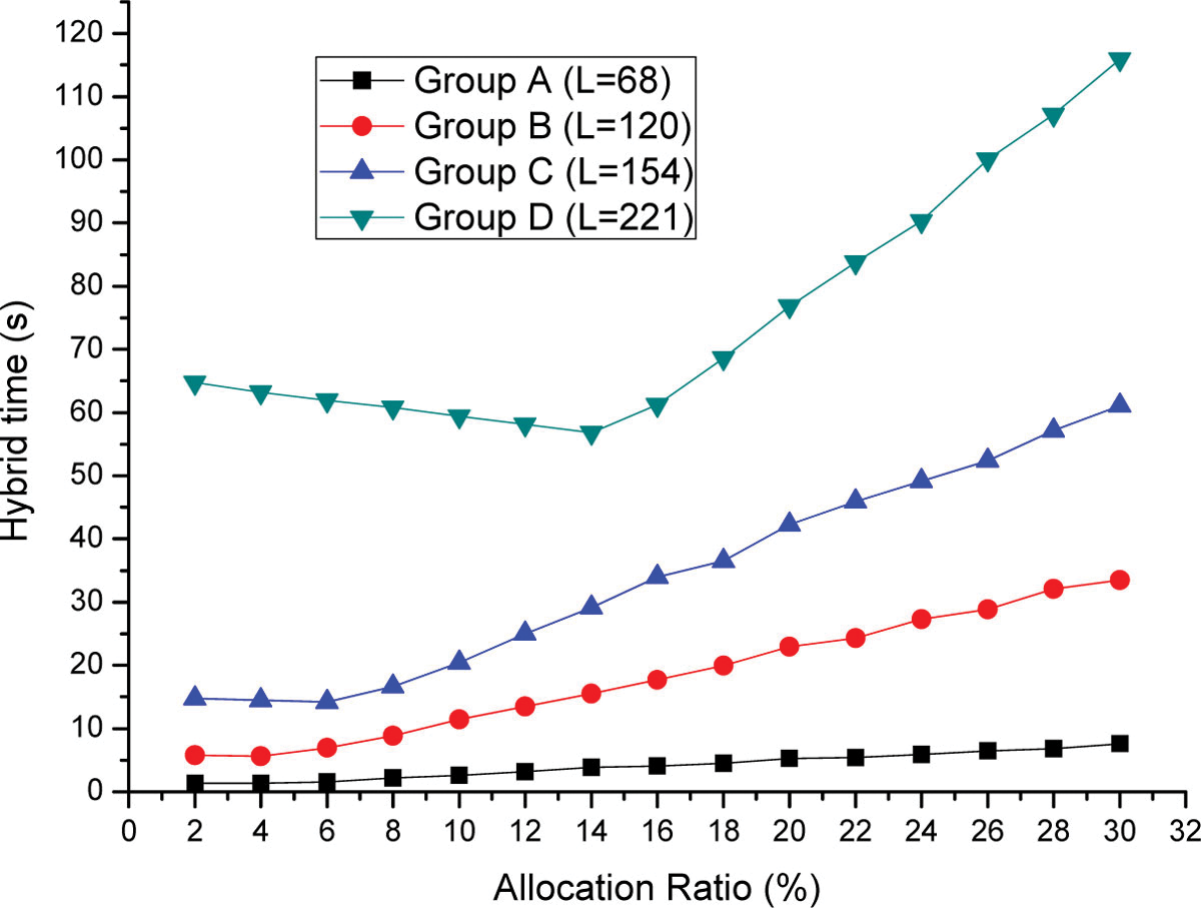
**Experimental results of execution time of hybrid system for four different sequence groups.** This figure describes the experimental results of execution time of hybrid system for four different sequence groups when task allocation ration increases from 2% to 30%. According to this figure, the minimal execution time of the hybrid system and the corresponding optimal allocation ratio are indicated.

Hybrid system execution time measurements for different allocation ratios ranging from 2% to 30% with 2% increasing step are shown in Figure 7. The optimal allocation ratios for minimal execution time in the groups A, B, C, and D are approximately 4%, 4%, 6%, and 14%, respectively. The estimated allocation ratio of the groups A, B, and C are slightly far from the real optimal value, mainly because the average CPU and GPU execution time is not estimated accurately. The error is acceptable because for short sequences, the execution time is very close to that of the optimal allocation ratio. For the group D with longer sequence, the optimal allocation ratio is very close to the estimated one. It can be inferred that our allocation method based on the average CPU or GPU execution time is reasonable. In group D, over 14% of computation task is allocated for CPU processing, which utilizes the CPU processing ability a lot.

#### Hybrid system speedup

Hybrid system execution time is measured for each group. To compare with CPU-only and GPU-only implementations, execution time on the CPU and GPU platforms where all computation tasks are loaded onto these devices is also obtained. The execution time and speedup are listed in Table 4. For the sequences in the group of B, speedup of 15.42× and 15.93× over the same optimized quad-core CPU implementation is achieved for the GPU and the hybrid system respectively. Speedup of over 50× over single Xeon E5620 core can also be achieved for this group. For group D, speedup of 5.83× and 6.75× over CPU-only implementation is achieved for both systems, showing the hybrid system to be 1.16× faster than that of GPU-only implementation. It means that our hybrid system can have 16% performance advantage over GPU-only implementation for Zuker algorithm applications for this group of sequences.

**Table 4:** Experimental results of execution time (s) and speedup (Sp) on different platforms for four sequence groups

Opt. platforms	A (L = 68)		B (L = 120)		C (L = 154)		D (L = 221)	
	
	Time	Sp.	Time	Sp.	Time	Sp.	Time	Sp.
Opt. quad-CPU	20.248	1	90.370	1	163.612	1	380.824	1
Opt. GPU	1.360	14.89	5.860	15.42	15.080	10.85	65.400	5.83
Hybrid system	1.280	15.82	5.673	15.93	13.870	11.80	56.386	6.75

## Discussion

According to the results of Table 4, the speedup over CPU-only implementation achieved for the hybrid system for the group B is larger than the rest of the groups. It can be inferred that the the efficiency of the hybrid system can be exploited well for sequences in this group. For longer sequences, the speedup is decreasing. It is mainly because that different portions of the Zuker algorithm have different computational complexity and GPU efficiency [19]. For longer sequences in group D, the GPU efficiency is becoming lower. The speedup of GPU over CPU in group D is 5.83×, lower than that of the rest of groups. We can infer that although the main idea of the proposed hybrid system is to exploit CPU computing ability, the performance of the hybrid system is still limited to that of GPU. In group D, a majority of sequences (over 85%) are still processed on GPU, which is the primary computing platform in the hybrid system. The key point of the hybrid system is the task allocation between the CPU and GPU. The proposed task allocation scheme may only be used to multiple sequences with same or similar length. For these sequences, we measure the estimated average execution time of each sequence on both CPU and GPU platform to calculate the speedup of GPU over CPU. The speedup then can be used to estimated the boundary value B for allocation of tasks between CPU and GPU. According to the boundary value B, the input numbered sequences are divided into two parts which are sent to CPU and GPU for parallel processing respectively. Although we only evaluated the hybrid accelerating method in the testbed with one CPU and one GPU, the proposed method can be easily employed to hardware platforms with multiple CPUs and GPUs. In these systems, the performance of multiple CPUs and GPUs must be estimated to instruct the task allocation, which will become a little complicated. Take the hardware system with two CPUs and GPUs for example, there may be three boundary values to be calculated to determine how many sequences will be sent to multiple CPUs and GPUs respectively.

## Conclusions

The Zuker algorithm is widely used for RNA secondary structure prediction. Based on careful investigation of the CPU and the GPU architecture, a novel CPU-GPU hybrid accelerating system for Zuker algorithm applications is proposed in the current study. Performance differences of CPU and GPU in the task allocation scheme is considered to obtain the workload balance. To improve the hybrid system performance, implementations of the Zuker algorithm on both the CPU and GPU platforms are optimized. The experimental results show that the hybrid accelerating system achieves a speedup factor of 15.93× and 16% performance advantages over optimized multi-core CPU and GPU implementations respectively. Moreover, more than 14% computation task is executed on CPU. The method combining CPU and GPU to accelerate the Zuker algorithm is proven to be promising and can also be employed to other bioinformatics applications.

## Competing interests

The authors declare that they have no competing interests.

## Authors' contributions

Guoqing Lei carried out the hybrid Zuker computing system, participated in the characteristics analysis of the Zuker algorithm and drafted the manuscript. Yong Dou conceived of the study, and participated in its design and helped to draft the manuscript. Wen Wan participated in the analysis of original ViennaRNA-1.8.4 software package, GPU implementation and performance measurement. Fei Xia helped to analyze the Zuker algorithm and instructed the parallel implementation. Rongchun Li participated in the implementation of SSE instructions and correctness verification. Meng Ma, Dan Zou participated in the hybrid system implementation. All authors read and approved the final manuscript.
